# Towards a Semi-Automatic Early Warning System for Vector-Borne Diseases

**DOI:** 10.3390/ijerph18041823

**Published:** 2021-02-13

**Authors:** Panagiotis Pergantas, Nikos E. Papanikolaou, Chrisovalantis Malesios, Andreas Tsatsaris, Marios Kondakis, Iokasti Perganta, Yiannis Tselentis, Nikos Demiris

**Affiliations:** 1 Bioapplications Ltd., 30 Ioannou Perganta Str., 32100 Levadia, Greece; pergantas@bioapplications.gr (P.P.); iokasti2001@gmail.com (I.P.); 2 Laboratory of Agricultural Zoology and Entomology, Department of Crop Science, Agricultural University of Athens, 75 Iera Odos Str., 11855 Athens, Greece; 3 Directorate of Plant Produce Protection, Greek Ministry of Rural Development and Food, 150 Sygrou Ave., 17671 Athens, Greece; 4 Department of Agricultural Economics and Rural Development, Agricultural University of Athens, 75 Iera Odos Str., 11855 Athens, Greece; malesios@aua.gr; 5 Department of Surveying and Geoinformatics Engineering, University of West Attica, 28 Ag. Spiridonos Str., 12243 Egaleo, Athens, Greece; atsats@uniwa.gr; 6 Department of Statistics, Athens University of Economics and Business, 76 Patision Str., 10434 Athens, Greece; kondakis@gmail.com (M.K.); nikos@aueb.gr (N.D.); 7 Regional Public Health Laboratory, Faculty of Medicine, University of Crete, 13 Andrea Kalokerinou Str., 71500 Giofirakia, Greece; yannis.tselentis@gmail.com

**Keywords:** mosquitoes, malaria, basic reproduction number

## Abstract

The emergence and spread of vector-borne diseases (VBDs) is a function of biotic, abiotic and socio-economic drivers of disease while their economic and societal burden depends upon a number of time-varying factors. This work is concerned with the development of an early warning system that can act as a predictive tool for public health preparedness and response. We employ a host-vector model that combines entomological (mosquito data), social (immigration rate, demographic data), environmental (temperature) and geographical data (risk areas). The output consists of appropriate maps depicting suitable risk measures such as the basic reproduction number, *R*_0_, and the probability of getting infected by the disease. These tools consist of the backbone of a semi-automatic early warning system tool which can potentially aid the monitoring and control of VBDs in different settings. In addition, it can be used for optimizing the cost-effectiveness of distinct control measures and the integration of open geospatial and climatological data. The R code used to generate the risk indicators and the corresponding spatial maps along with the data is made available.

## 1. Introduction

Vector-borne diseases (VBDs) are caused by parasites, viruses or bacteria that are transmitted by a vector [1]. Mosquitoes, ticks, sandflies, triatomine bugs, tsetse flies, fleas, black flies, aquatic snails and lice are known to act as vectors of this kind of disease [2]. The emergence and spread of VBDs in Europe is a function of biotic, abiotic and socio-economic drivers of disease [3]. Public health decision-making generally requires early warning output from frameworks that are based on uncertain information [4,5]. Globalization and climate changes, along with certain socio-demographic determinants can be critical drivers of VBDs. Hence, observing changes in these drivers can help envision, or even predict, fluctuations in many infectious diseases [3,6].

Among all the hematophagous arthropod vectors, mosquitoes (Diptera: Culicidae) are the leading vector of several human diseases. Mosquito-borne diseases (MBDs) include malaria, West Nile virus, chikungunya and Zika virus [7,8]. Blood meals are necessary for mosquito females as the protein source to produce and mature their eggs [9]. Τhis activity allows mosquitoes to transmit several pathogens and parasites, becoming extremely important insects for human health [7]. Taking into account the invasive success of several mosquito species due to worldwide human mobility and trade [10], the development of early warning and control methods seems imperative [11].

Tropical VBDs such as malaria have attracted much attention due to their impact on human health [12,13]. Malaria is, perhaps, the most well-studied VBD in terms of its transmission dynamics and the potential for adaptation. It is known that five species of *Plasmodium* cause disease in humans [14]. According to the 2019 World malaria report, an estimated 228 million cases of malaria occurred worldwide [15]. Malaria is endemic in more than 100 countries around the world, mainly in sub-Saharan Africa and Asia, and is transmitted through the bite of the infected female *Anopheles* mosquito. 

Considering the importance of vector-borne diseases in human health, it is imperative to work towards creating a suitable framework for an early warning system that would improve our understanding of the connectivity between existing and potential vector-borne risk areas. In this study, we focus upon malaria in Greece, a previously endemic disease that was eradicated in the 1950s by coordinated efforts of the WHO and the local authorities. Sporadic local *P.* *vivax* malaria outbreaks during the last decade indicate that targeted vector control remains imperative for preventing re-emergence. We firstly present a host-vector spatially explicit model which integrates entomological, geographical, social and environmental evidence from 12 municipalities of the prefecture of central Greece (see Table A1 in Appendix A for a description of the population and density information of the municipalities in the study), in order to guide the mosquito control efforts. To this end, we focused on the estimation of parameters related to the *Anopheles’* population dynamics and the corresponding risk measures derived from them. In addition, we presented the basis for an appropriate (semi)-automated, open-source, early warning tool based upon the above estimates of suitable risk indicators. The ultimate goal of this paper in terms of public health would be to use the proposed model, as well as the corresponding spatial mapping of the derived estimates from this model, as an early warning system with meteorological inputs, thus facilitating improved decision making and disease prevention. The R code calculating the risk measures and depicting them on spatial maps of this risk is made freely available on GitHub.

## 2. Methods

### 2.1. Study Area and Data Collection

**Figure 1:**
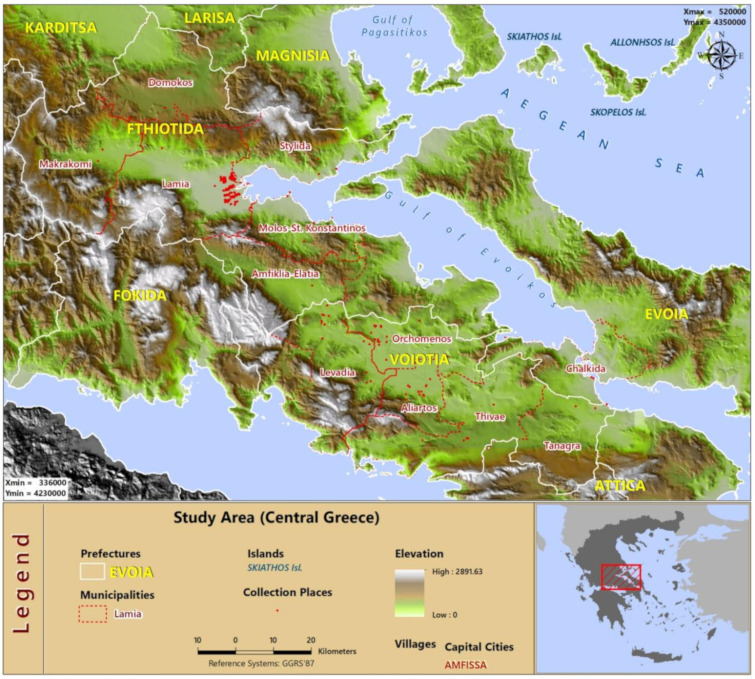
Map of study area.

The study took place in 12 municipalities of the prefecture of central Greece (Figure 1). The study area was about 406.000 ha.

Considering mosquito data collection, we used CO_2_ traps. Therefore, our data consisted of mosquito female adults, which are the malaria vectors. The traps were set up in 10 areas, consisting of a total of 393 sample-collecting stations in the outskirts of towns and villages of the prefecture of central Greece (see Figure 1 for a visual inspection of all collection places). Breeding sites included canals, rice pads, tanks, etc., most of them previously checked for the presence of mosquitoes using drones. The traps were placed and checked every 10 to 15 days between 6 March 2018 and 29 August 2018. Genus identification was performed using the morphological examination of the mosquito samples. The species *Anopheles sacharovi*, *An. maculipennis*, *An. superpictus*, *An. claviger* and *An. hyrcanus* are the most important species in the study region, accounting for approximately 90% of all *Anopheles* species in the region [11]. We, therefore, suppose that all the positive samplings for *Anopheles* mosquitoes belong to species that are malaria vectors.

Temperature data were recorded from the meteorological stations of the National Observatory of Athens (NOA). There were 10 meteorological stations of the NOA recording temperature data at the study area. For our modeling, we utilized the average temperature values with a 30-day interval, starting from the first Saturday of each month, between the March and August of 2018. The inverse distance weighting (IDW) interpolation method [16] was additionally used to estimate the average temperatures at the locations where no measurements were available.

### 2.2. The Spatial Predictive Model

In this paper, we present a host-vector model that combines entomological, social, environmental and geographical data to provide estimates on the average infection number due to malaria in central Greece (Table A2). The model has been already presented [11] and here we provide a short summary. In addition to the standard entomological parameters which are used within the well-established Ross-–Macdonald mathematical model [17], our model takes into account the potential host population in the central Greece region, related to migrants from regions endemic to malaria [18]. Their prevalence in terms of the *P. vivax* is determined from the latest World Malaria Report of the WHO [19].

The transmission risk measures obtained by the model are the basic reproduction rate of the disease, namely $R_{0}$, which represents a natural threshold parameter appropriate for disease control since an outbreak may occur only when $R_{0}>1$. This is indicative of the potential for disease spread should one infective individual starts an outbreak. In addition, we estimate the probability of an individual getting infected, τ=Pr(infection), and the number of expected infections, say E(infections) given by:E(infections)=Pr(infection)×(# of susceptibles). Those two risk measures are conditional upon an outbreak taking off and are only meaningful in the event of introducing infected individuals in the area, as is $R_{0}$.

We denote with ${\hat{R}}_{0}$,$\hat{\tau }$ the corresponding model-based point (typically median) estimates of these measures and we calculate local $R_{0}$ estimates for each sample-collecting station using:

(1) $${\hat{R}}_{0i}=\frac{Vec_{i}\cdot b_{i}\cdot c}{r_{i}},$$

where Vec is the vectorial capacity, that is, the expected number of infective mosquito bites that would eventually arise from all the mosquitoes that would bite a single fully infectious person on a single day [20] and is given by:

(2) $$Vec_{i}=\frac{m_{i}\cdot \alpha_{i}^{2}\cdot \exp\left(-g_{i}\cdot v_{i}\right)}{g_{i}}$$

In (1) and (2), $m_{i}$ denotes the numbers of mosquitoes in each station i; $\alpha_{i}$ the biting rate, that is, the percentage of mosquitoes that feed on humans each day; $b_{i}$ the probability a bite produces infection to a human; $r_{i}$ the average daily recovery rate per day; $v_{i}$ the mosquito latent period, that is, the number of days from infection to infectiousness; $g_{i}$ the mosquito mortality rate per day. Especially parameter $g_{i}$ has been shown in previous research [20] to be dependent on the temperature levels, hence we also utilize recorded and/or interpolated temperatures for our calculations. Finally, with c we denote the probability a bite turns a susceptible mosquito to infected, which for our analysis is set to the constant value of 0.5. The parameters $\alpha_{i}$, $b_{i}$, $r_{i}$ and $v_{i}$ were sampled from suitable distributions according to the relevant literature [17,20,21], whereas $g_{i}$ changes with the temperature levels [20], which are currently represented by monthly means of temperature [11]. An analytical description of parameters and relevant distributions utilized for the calculations of risk measures is included in Table A3 in Appendix A. 

In addition to the entomological part of the proposed model, estimation of the external host component due to the migration is embedded into the risk parameter calculations, by utilizing an exponential kernel function, $W_{ik}$, (*k* = 1,2,3) of the form:

(3) $$W_{ik}=\theta \cdot \exp\left(-\theta \cdot d_{ik}\right)$$

$W_{ik}$ is used to model the spatial part of the potential hosts, with θ being a weight factor. In (3) $d_{ik}$ denotes the distances from larvae areas, measured during the three periods of potential hosts’ monitoring, *k*. Subsequently, the estimation of the external host component due to the migration is approximated by ${\hat{\mu }}_{0i}=\sum_{k=1}^{3}\mu_{0ik}\cdot W_{ik}.$ This estimated proportion of initially infected hosts, ${\hat{\mu }}_{0i}$, is then multiplied by a pre-specified incidence rate. A deterministic sensitivity analysis was conducted [11] and the overall qualitative picture remained unchanged although the quantitative scale varied somewhat. 

Finally, estimation of $\hat{\tau }$, should a local outbreak is initiated, is performed by solving the non-linear equation:

(4) $$1+{\hat{\mu }}_{0i}-{\hat{\tau }}_{i}-\exp(-{\hat{\tau }}_{i}\cdot {\hat{R}}_{0i})=0$$

which only applies for ${\hat{R}}_{0i}\geq 1$. We set ${\hat{\tau }}_{i}=0$ when ${\hat{R}}_{0i}<1$.

### 2.3. Towards a Semi-Automatic Early Warning System Tool

The semi-automatic early warning system tool was developed using the open-access R software environment [22], which is free and compatible with most common programming languages. In particular, R was used for all the steps of the process, including constructing $R_{0}$ and other risk measure maps. Similar maps, in the case of other infectious viruses, have been presented elsewhere [23]. A large collection of static maps from various online sources (e.g., Google Maps provided by Google LLC in California, USA and Stamen Maps provided by data visualization and cartography design studio in San Francisco, USA) can be used within R for the effective visualization of the parameters of interest.

**Figure 2:**
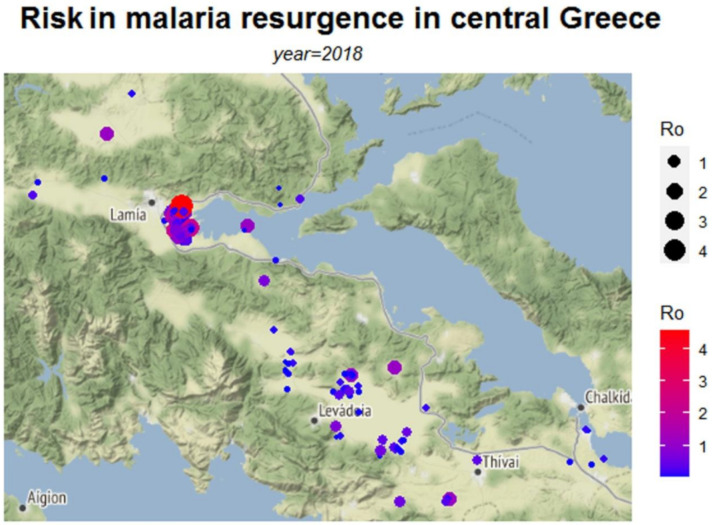
Map depicting the risk of malaria transmission in the form of *R_0_*.

**Figure 3:**
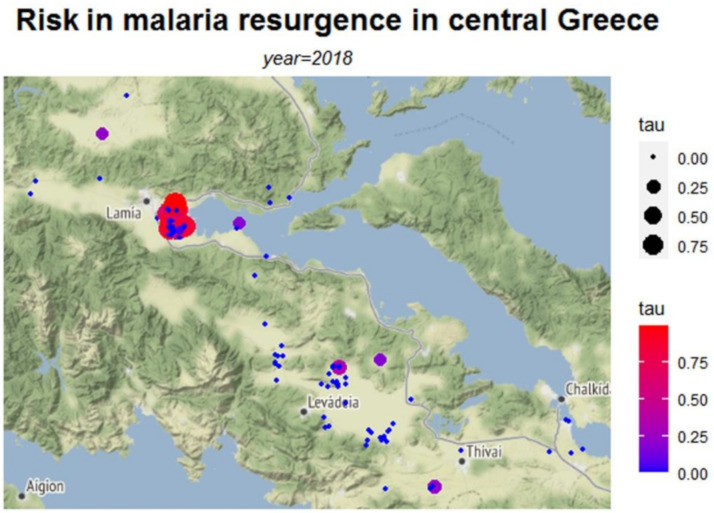
Map of risk of malaria transmission, computed by the EWS model (*τ* estimates).

**Figure 4:**
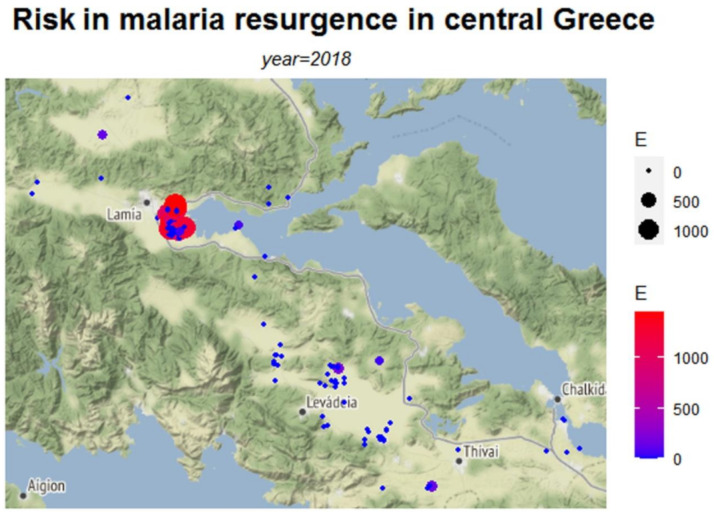
Map of risk of malaria transmission, computed by the EWS model (*Ε* estimates).

**Figure A1:**
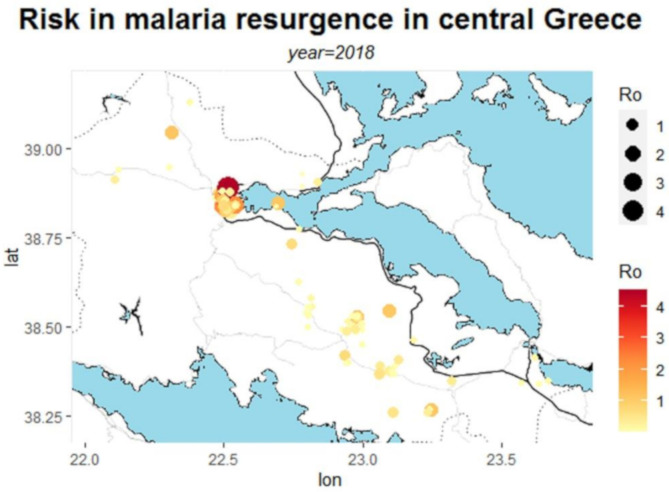
Point estimate map of risk of malaria transmission (*R_0_* estimates).

**Figure A2:**
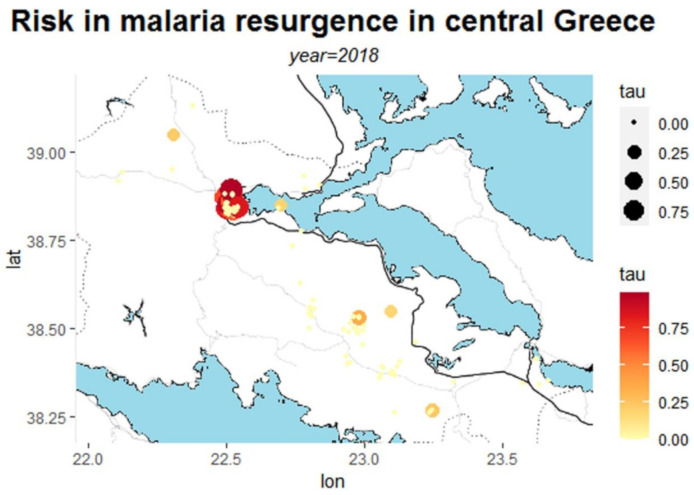
Point estimate map of risk of malaria transmission (*τ* estimates).

**Figure A3:**
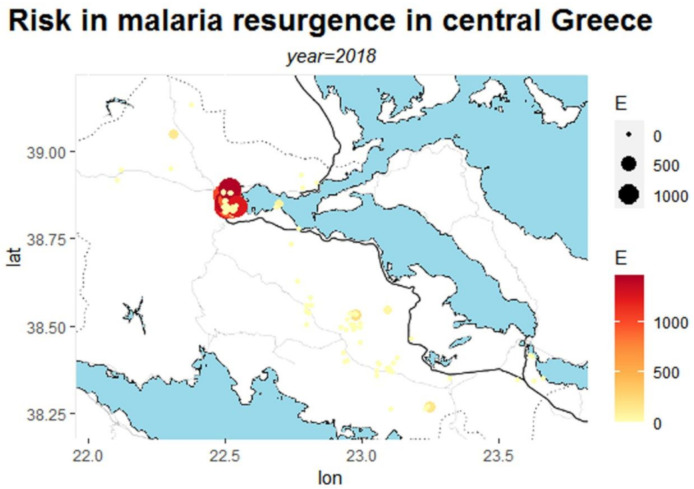
Point estimate map of risk of malaria transmission (*E* estimates).

**Figure A4:**
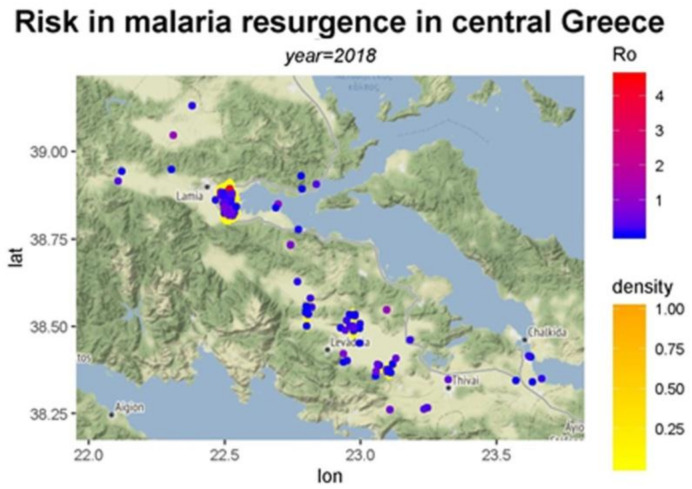
Heat-map of risk of malaria transmission (R_0_ estimates).

**Figure A5:**
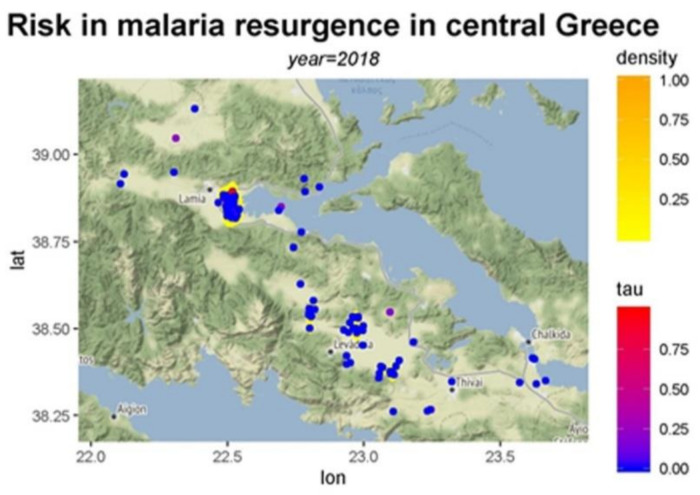
Heat-map of risk of malaria transmission (*τ* estimates).

**Figure A6:**
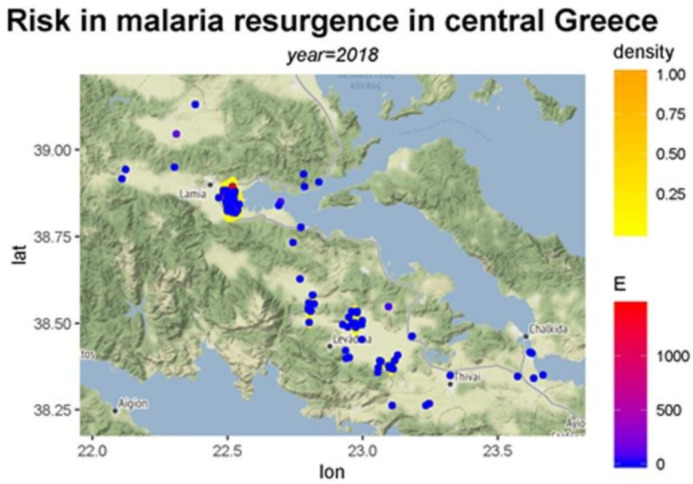
Heat-map of risk of malaria transmission (*E* estimates).

The calculations were facilitated using a variety of software packages. Specifically, the *dplyr* package is used to make data frame manipulation in an efficient way and the *ggmap* package [24] provides the main methods for accessing and downloading Google and Stamen maps as well as for generating the data graphs of this work. Furthermore, the tools used to construct the graphs presented here can be separated into two main categories by graph form. The first category includes the *qmplot* function which makes a quick overview of maps along with data points. Herein, the data points are spatial points that reflect the measurements upon both terrain-background based maps (Figure 2, Figure 3 and Figure 4) and toner-background based maps (Figure A1, Figure A2 and Figure A3) and vary in size and in color according to the values of (*R*o), (*τ*) and (*E*) respectively. The second category includes the *stat_density2d* function which performs a two-dimensional kernel density estimation with an axis-aligned bivariate normal kernel function (Figure A4, Figure A5 and Figure A6). This density function creates a continuous surface by measuring the contribution of each data point on a map area. This contribution is smoothed out from a single point into a region of space surrounding the point. The kernel density estimate evaluated on a grid is given by:

(5) $$\hat{f}(x,y)=\frac{\sum_{i}\phi \left(\frac{x-x_{i}}{\sigma_{x}}\right)\cdot \phi \left(\frac{y-y_{i}}{\sigma_{y}}\right)}{n\cdot \sigma_{x}\cdot \sigma_{y}}$$

where density φ is the standard normal distribution and $\mathrm{diag}\left(\sigma_{x}{}^{2},\sigma_{y}{}^{2}\right)$ is the bandwidth diagonal matrix which controls the amount and orientation of smoothing induced. The bandwidth plays the role of the covariance matrix of the bivariate normal kernel [25]. The output surface shows where point features are concentrated, by measuring the accumulated intersections of the individual areas. The way of density calculation depends on the bandwidth that uses a default search radius. In addition, the *bins* parameter is a control parameter that defines the number of contour levels. We used 100 bins for the construction of Figure A4, Figure A5 and Figure A6. Code for implementing all the methods in the paper is provided at https://github.com/valadis/EWS_spatial_maps_R_code.git.

## 3. Results

In Figure 2, Figure 3 and Figure 4, the risk maps for the risk parameters of interest are depicted (Table A4 in Appendix A presents descriptive statistics of the estimated risk measures). These maps are based on the coordinates of the data points with the intensity of each risk measure varying with color. 

The risk map in Figure 2 depicts the risk as expressed by the median basic reproduction number *R*_0_ for each area, whereas Figure 3 presents the estimated probability of getting infected, *τ*. Figure 4 shows the number of expected infections *E*, as estimated by the spatial predictive model.

These visualization maps were created using the *ggmap* function of ggmap package. Alternative presentations based on other functions such as the *qmplot* option are also available in the ggmap package. Appendix A includes alternative visualizations of our results, including maps of point estimates using alternative terrain representations (Figure A1, Figure A2 and Figure A3) or heat-maps (Figure A4, Figure A5 and Figure A6) and different options may suit different users based upon their needs.

Values of the *R*_0_ above 1 (Figure 2), indicate where the greatest potential for risk is located. The highest risk is primarily located in the area of Lamia, with *R*_0_ reaching values as high as 4. The probability of getting infected from low to high (zero to 0.75) was depicted in the map of Figure 3 and the Lamia region was found to have the highest risk. However, the map also suggests the non-negligible probability of infection (*τ* estimates between 0.25 and 0.5) in the largely dispersed rural areas of the prefecture. As these rural areas have low populations, the expected number of infections is relatively low as shown in Figure 4, revealing the complementary characteristics of the different risk measures. The highest number of potential infections is concentrated in the wider region of Lamia. 

**Figure 5:**
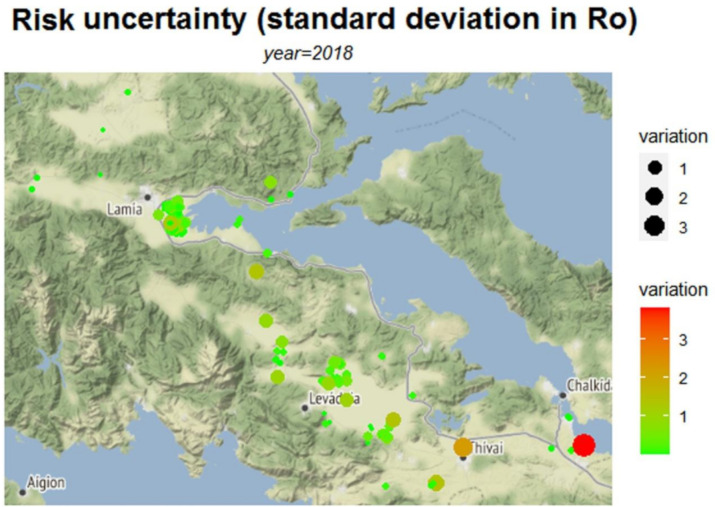
Uncertainty in *R*_0_, in the form of the standard deviation of *R*_0_ (based on 1000 samples).

In addition to the risk maps based upon the estimated parameters such as the basic reproduction number *R*_0_, our approach enables the presentation of the corresponding uncertainty of the estimated parameters (or functions thereof) by way of depicting the associated variability, for example, via the variance or standard deviation. For example, the risk map of malaria transmission based on *R*_0_ (Figure 2) can naturally be combined with a variability map of the parameter (see Figure 5) in order to provide a more robust tool for the monitoring of transmission of VBDs. This type of combined reporting can be potentially applied to the other measures of risk assessment. Perhaps more importantly, it reveals knowledge gaps since high uncertainty suggests that further sampling is required in those areas in order to reduce this variability.

## 4. Discussion

Our study introduces a model-based framework that integrates entomological, geographical, social and environmental evidence in order to examine the potential for malaria resurgence in Greece. This results towards a semi-automatic open-source tool that can be used for the monitoring and early warning of mosquito-borne diseases, such as malaria and West Nile virus. The early warning system takes into account the spatial distribution of risk via suitable mathematical modeling to generate appropriate maps that can adequately describe the spatial variation in risk.

The results suggest that five to six distinct risk areas in the spatial maps with potential for malaria resurgence can be identified by inspecting the generated graphs. Specifically, the areas of higher risk are those close to the municipality of Lamia as expected due to the local rice fields and to a lesser extent in the lowlands around Levadia and Thivae municipalities wherever the obsolete irrigation system is responsible for a large number of *Anopheles* mosquito breeding sites. Generally, the risk of malaria resurgence is greatly facilitated by the coexisting of people from countries where malaria is endemic and who are engaged in agricultural work in areas where there are Anopheles mosquito breeding habitats such as areas with paddies (e.g., municipality of Lamia) and irrigation canals. These areas can serve as hot spots for the resurgence of malaria. 

Considering VBDs as an emerging public health threat worldwide [6,14], we expect our methodology to have a bearing on the evaluation of the epidemic risks of such diseases in a given area. Recently, refugee populations are being hosted throughout Europe, including Greece [26], potentially increasing the risk of vector-borne disease transmission. In addition, several disease vectors in refugee camps in Greece have been recorded [27], indicating a possible risk factor for disease transmission. An early warning system for vector-borne diseases of the sort presented in this paper could be suitable for preventing disease spread.

Our approach could be also used as a tool for the efficient control of mosquito species, indicating time control periods, preventing their expansion and, therefore, the potential for disease transmission. Understanding the mosquito spatial distribution is of importance for public health and a cornerstone for studies aiming to understand their expansion [10,28,29,30,31]. Studies dealing with species distribution models may set aside important factors that drive these models such as the quality of the training data, as well as critical abiotic factors [10]. Our mosquito sampling method consisted of CO_2_ traps. Consequently, our data involved only female *Anopheles* individuals which serve as vectors of malaria, so that we overcame issues of sex-biased data that may arise by larvae sampling. 

Our model accounts, in turn, for temperature fluctuations, which are particularly important in insect performance [32,33,34]. Temperature is the main abiotic factor that determines insect distribution, affecting critical aspects of their life history such as development, survival, reproduction and life span [35,36,37]. This results in a further effect on insect fitness, determining their population dynamics [38,39,40]. Therefore, via accounting for temperature fluctuations our model is tailored towards the precise estimation of mosquito population growth.

Our method has some limitations. It relies on detailed evidence of various types. Data of this kind may or may not be readily available and some sort of approximation is often used. However, such issues are reflected in the uncertainty of the risk measures and the corresponding maps are a natural by-product of the proposed tool. In fact, these maps offer an opportunity since they suggest where additional sampling should occur in order to reduce this uncertainty. In addition, most such data are observational data and do not form part of a randomized controlled experiment. This study represents a typical example, and appropriate counterfactual scenarios are common in the area, including what would have happened had no vector control method been applied. These scenarios are based on established theory though and including the corresponding uncertainty facilitates scientifically honest reporting and leads to additional data collection as described above.

## 5. Conclusions

The threat of a rapidly changing planet includes precarious spatial-temporal change dynamics associated with VBDs [41]. For example, disease transmission may vary strongly and unimodally with temperature [6]. This poses new conceptual and practical challenges in relation to sustainable health resilience, and therefore timely control of VBDs may act towards this direction. We expect that our attempt towards a semi-automatic early warning system tool we developed can potentially aid the monitoring and control of VBDs in different settings. In addition, it can be used for optimizing the cost-effectiveness of distinct control measures and the integration of open geospatial and climatological data. Perhaps more importantly, this system can enhance our ability to predict the risk of disease outbreaks in different climatic conditions. The developed methods can be adapted for usage in countries with similar vector-borne disease potential. To this end, the R code producing and depicting the risk indicators is provided in the supplement.

## Data Availability

The data presented in this study are openly available in https://github.com/valadis/EWS_spatial_maps_R_code.git.

## Appendix A

Alternative representations of risk through spatial maps in R.

**Table A1:** Population and density data on the 12 municipalities involved in the current study.

Municipality	Area	Population	Population Density	Urban Area
Aliartos	258,449,230.96	11,645	45.06	10,329,054
Amfiklia-Elatia	533,074,049.91	11,902	22.33	7,157,762
Domokos	708,266,142.17	15,227	21.50	3,088,636
Thivae	832,281,806.11	34,401	41.33	16,878,756
Lamia	946,708,662.20	71,006	75.00	27,276,528
Levadia	690,056,941.60	33,425	48.44	11,000,844
Makrakomi	837,249,336.29	19,211	22.95	6,073,913
Molos-St. Konstantinos	339,219,708.58	13,048	38.46	8,691,250
Orchomenos	418,237,273.08	13,107	31.34	10,791,795
Stylida	462,005,680.14	13,851	29.98	6,335,263
Tanagra	460,288,037.47	15,086	32.78	32,420,422
Chalkida	425,298,043.46	86,393	203.14	56,371,907

**Table A2:** Data used in the study.

Data Type	Detail	Source	References
Entomological	Number of mosquitoes	Trap survey	This study
Social	Number of immigrants	Information collected by Bioapplications Ltd.	https://www.bioapplications.gr/?lang=en (accessed on 6 December 2020)
Environmental	Temperature	Meteorological stations (National Observatory of Athens)	https://www.meteo.gr/meteomaps/ (accessed on 6 December 2020)
Geographical	Maps	National Bank of Meteorological and Hydrographic Data (Figure 1)	www.hydroscope.gr (accessed on 6 December 2020)
		R Package: ggmap using Stamen open-source maps (Figure 2, Figure 3, Figure 4 and Figure 5, Figure A1, Figure A2, Figure A3, Figure A4, Figure A5 and Figure A6)	maps.stamen.com (accessed on 6 December 2020)

**Table A3:** List of parameters utilized for the calculation of risk measures and their interpretations along with corresponding references.

Parameter or Variable	Interpretation	Distribution/Value	Reference
$\alpha_{i}$	% of mosquitoes that feed on humans/day	Uniform (0.01, 0.5)	[17,20,21],
$b_{i}$	Probability a bite produces infection to a human	Uniform (0.2, 0.5)	[17,20,21]
c	Probability a bite turns a susceptible mosquito to infected	0.5	[17,20,21]
$r_{i}$	Average daily recovery rate/day	Uniform (0.01, 0.5)	[17,20,21]
$v_{i}$	# of days from infection to infectiousness in the mosquito	Uniform (5, 15)	[17,20,21]
$m_{i}$	The ratio of mosquitoes to humans in region *i*	Observed data	
$g_{i}$	Mosquito mortality rate/day	$g_{i}=f\left(T_{i}\right)$	[20]
$T_{i}$	Average temperatures	Observed data	
θ	Spatial parameter for modeling the spatial component of migrant transmission	Observed data	
$d_{ik}$	Distances of migrant population from the larvae areas	Observed data	

**Table A4:** Descriptive statistics of the estimated risk measures, broken down by Municipality.

Municipality		Ro	τ	E	Variation of Ro
**Aliartos**	Mean	0.162	0	0	0.125
	Median	0.048	0	0	0
	Std. Deviation	0.190	0	0	0.342
	Minimum	0.010	0	0	0
	Maximum	0.563	0	0	1
Amfiklia-Elatia	Mean	0.071	0	0	1
	Median	0071	0	0	1
	Std. Deviation		0	0	
	Minimum	0.071	0	0	1
	Maximum	0.071	0	0	1
Chalkida	Mean	0.004	0	0	0
	Median	0.004	0	0	0
	Std. Deviation		0	0	0
	Minimum	0.004	0	0	0
	Maximum	0.004	0	0	0
Domokos	Mean	0.602	0.110	69,400	0
	Median	0.602	0.110	69,400	0
	Std. Deviation	0.741	0.155	98,146	0
	Minimum	0.078	0	0	0
	Maximum	1.126	0.220	138,800	0
Lamia	Mean	0.258	0.033	49,316	0.035
	Median	0.096	0	0	0
	Std. Deviation	0.474	0.148	218,071	0.185
	Minimum	0.003	0	0	0
	Maximum	4.55	0.989	1,458,600	1
Levadia	Mean	0.111	0	0	0.188
	Median	0.036	0	0	0
	Std. Deviation	0.184	0	0	0.403
	Minimum	0.007	0	0	0
	Maximum	0.729	0	0	1
Makrakomi	Mean	0.161	0	0	0
	Median	0.161	0	0	0
	Std. Deviation	0.191	0	0	0
	Minimum	0.026	0	0	0
	Maximum	0.296	0	0	0
Molos-St. Konstantinos	Mean	0.381	0.037	13,960	0.2
	Median	0.061	0	0	0
	Std. Deviation	0.492	0.083	31,216	0.447
	Minimum	0.011	0	0	0
	Maximum	1.105	0.185	69,800	1
Orchomenos	Mean	0.172	0.019	9146	0.143
	Median	0.062	0	0	0
	Std. Deviation	0.303	0.073	39,138	0.356
	Minimum	0.005	0	0	0
	Maximum	1.228	0.353	201,900	1
Stylida	Mean	0.098	0	0	0.333
	Median	0.011	0	0	0
	Std. Deviation	0.153	0	0	0.577
	Minimum	0.009	0	0	0
	Maximum	0.274	0	0	1
Tanagra	Mean	0.054	0	0	1.333
	Median	0.026	0	0	0
	Std. Deviation	0.057	0	0	2.309
	Minimum	0.016	0	0	0
	Maximum	0.119	0	0	4
Thivae	Mean	0.492	0.048	32,040	0.6
	Median	0.385	0	0	0
	Std. Deviation	0.384	0.107	71,644	0.894
	Minimum	0.118	0	0	0
	Maximum	1.129	0.239	160,200	2
Total	Mean	0.243	0.029	40,491	0.076
	Median	0.085	0	0	0
	Std. Deviation	0.443	0.134	194,946	0.326
	Minimum	0.003	0	0	0
	Maximum	4.550	0.989	1,458,600	4
